# Prevalence and Associated Factors of Subjective Halitosis in Korean Adolescents

**DOI:** 10.1371/journal.pone.0140214

**Published:** 2015-10-13

**Authors:** So Young Kim, Songyong Sim, Sung-Gyun Kim, Bumjung Park, Hyo Geun Choi

**Affiliations:** 1 Department of Otorhinolaryngology-Head & Neck Surgery and Cancer Research Institute, Seoul National University College of Medicine, Seoul, Korea; 2 Department of Statistics, Hallym University, Chuncheon, Korea; 3 Department of Internal Medicine, College of Medicine, Hallym University, Chuncheon, Republic of Korea; 4 Department of Otorhinolaryngology-Head & Neck Surgery, Hallym University Sacred Heart Hospital, Anyang, Korea; University Hospital of the Albert-Ludwigs-University Freiburg, GERMANY

## Abstract

This study was conducted to estimate the prevalence and associated factors of subjective halitosis in adolescents. In total, 359,263 participants were selected from the Korea Youth Risk Behavior Web-based Survey (KYRBWS) from 2009 through 2013. Demographic data including age, sex, obesity and residency; psychosocial factors such as subjective health, stress, and economic levels; and dietary factors such as alcohol consumption; smoking; and fruit, soda, fast food, instant noodle, confection, and vegetable consumption were analyzed for correlations with halitosis using simple and multiple logistic regression analyses with complex sampling. In total, 23.6% of the participants reported the presence of halitosis. The following subjectively assessed factors were related to halitosis: poor health status (adjusted odds ratio [AOR] = 2.56), overweight or obese (AOR = 1.37), stress (AOR = 2.56), and lower economic levels (AOR = 1.85). The high intake of fast food (AOR = 1.15), instant noodles (AOR = 1.17), and confections (AOR = 1.17) and the low intake of fruits (AOR = 1.22) and vegetables (AOR = 1.19) were also related to halitosis. The prevalence of subjective halitosis in the studied adolescents was 23.6%. Specific psychosocial factors and dietary intake were related to halitosis.

## Introduction

Halitosis is defined as the presence of unpleasant or foul smelling breath that primarily originates from the oral cavity [1, 2]. Halitosis is common worldwide. Although the prevalence of halitosis varies according to the study population, definition and diagnostic tools, the prevalence of halitosis is approximately 10–30% in the general population [3–5].

Numerous origins or causes of halitosis exist. Halitosis can be classified as either primary halitosis, which originates from the exhalation by the lungs, or secondary halitosis, which relates with the mouth or upper airways [6]. Most secondary halitosis likely develops because of various foul smelling substances including volatile sulfur compounds (VSCs) in the oral cavity. The remainder of the extra-oral or systemic origins of halitosis encompass chronic sinusitis, post-nasal drip, nasal foreign bodies, respiratory infections, gastrointestinal conditions such as gastric reflux, diabetic ketoacidosis, renal failure, hepatic failure, certain rare metabolic diseases, such as diabetic ketoacidosis, and trimethylaminuria [7]. In addition to the above-mentioned pathological conditions, previous studies have reported several other factors that are associated with halitosis. Certain dietary components such as garlic, spicy food, tobacco, and alcohol may cause halitosis [8]. Moreover, demographic factors including age, sex, and psychosocial factors have also been reported to contribute to halitosis [9]. In pediatric populations, poor oral hygiene, female gender and older age were reported as related factors of halitosis [10, 11].

Halitosis in adolescents has several distinct features from that observed in adults or the elderly. Adult diseases that are known to be related to halitosis, such as diabetes, renal and hepatic failure, are relatively rare in adolescents. Moreover, adolescents have different lifestyles and environmental circumstances that may affect halitosis. For example, adolescents are more prone to exposure to and ingestion of junk food than adults are. Therefore, this population must be considered separately when identifying the factors associated with halitosis.

Thus far, a limited number of studies have examined halitosis in adolescents. Moreover, only a few studies have reported factors associated with halitosis including dietary, stress, and socio-economic factors, in addition to intra-oral, extra-oral, or systemic diseases. The present study was designed for a large representative adolescent population in Korea. To our knowledge, this study is the first regarding halitosis in a Korean adolescent population. We estimated the prevalence of halitosis in this population and comprehensively investigated the correlation of halitosis with many covariates of demographic, subjective health, stress, and economic factors, as well as of commonly encountered foods in daily life.

## Materials and Methods

### Study population and data collection

The Institutional Review Board of the Centers for Disease Control and Prevention of Korea (KCDC) approved this study (2014-06EXP-02-P-A). Written informed consent was obtained from each participant prior to the survey. Because this web based survey was performed at the school with huge participants, the informed consent from their parents was exempted. This consent procedure was approved by the IRB of KCDC.

This cross-sectional study used data from the Korea Youth Risk Behavior Web-based Survey (KYRBWS). This study covered the entire nation using statistical methods based on designed sampling and adjusted weighted value. Data from the KYRBWS conducted in 2009, 2010, 2011, 2012, and 2013 were analyzed. The data were collected by the KCDC. Korean adolescents in 7^th^ through 12^th^ grade completed the self-administered questionnaire voluntarily and anonymously. The validity and reliability of the KYRBWS has been documented by other studies [12, 13]. The surveys evaluated data from South Korean adolescents using stratified, two-stage (schools and classes) clustered sampling based on data from the Education Ministry. Sampling was weighted by statisticians, who performed post-stratification and who considered both the non-response rates and the extreme values.

Of 370,568 participants, we excluded the following participants from this study: participants who did not record their height or weight (11,303 participants), participants who did not fill out the educational level of their mothers (1 participant), and participants who did not fill out their alcohol consumption history (1 participant). Ultimately, 359,263 participants (184,801 males and 174,462 females) ranging in age from 12 to 18 years old were included in this study.

### Survey

Obesity was categorized into 4 groups according to the CDC guidelines regarding body mass index (BMI, kg/m^2^) for children and teens: obese ≥ 95^th^ percentile; overweight ≥ 85^th^ percentile and < 95^th^ percentile; healthy weight ≥ 5^th^ percentile and < 85^th^ percentile; underweight < 5^th^ percentile. The region of residence was divided into 3 groups by administrative district: large city, small city, and rural area. The subjective assessment of general health status was divided into 5 groups from very good to very bad. The stress levels of the participants were divided into 5 groups: severe, moderate, mild, a little, and no stress. Economic level was measured as 5 levels from the highest to lowest. The participants were asked to provide the number of days that they drank alcohol except in religious ceremony during the past month. Based on their responses, the participants were divided into 4 groups: 0 days a month, 1–5 days a month, 6–19 days a month, and ≥ 20 days a month. The participants were asked to provide the number of days that they smoked during the past month. Based on their responses, the participants were divided into 4 groups: 0 days a month, 1–5 days a month, 6–19 days a month, and ≥ 20 days a month. The participants were also asked the frequency of consuming fruit (not fruit juice), soda drink, fast food (such as pizza, hamburger, or chicken), instant noodles, confections, and vegetables during the past 7 days. Based on their responses, the participants were divided into 4 groups: ≥ 7 times a week, 3–6 times a week, 1–2 times a week, and 0 times a week. If the participants felt he or she had a subjective unpleasant odor originating from the mouth, this odor was recorded as subjective halitosis.

### Statistical analysis

The differences in general characteristics according to performance at school were calculated using a paired t-test for age, chi-square test for sex, and linear by linear association test for obesity; residence region; subjective health; stress level; economic level; alcohol consumption; smoking; and fruit, soda drink, fast food, instant noodle, confectionary, and vegetable intake frequencies.

Odds ratios for each possible halitosis risk factor were calculated by simple logistic regression analysis with complex sampling (unadjusted) and multiple logistic regression analysis with complex sampling. Two-tailed analyses were conducted, and P values lower than 0.05 were considered significant. Additionally, 95% confidence intervals (CIs) were calculated. After applying the weighted values recommended by the KYRBWS, all results are presented as weighted values. The results were analyzed statistically using SPSS ver. 21.0 (IBM, Armonk, NY, USA).

## Results

In total, 84,959 participants (23.6%) complained of halitosis. The mean age of the participants with halitosis was 15.1 years. Despite being significantly different, no clinically meaningful differences in age were observed between the halitosis and non-halitosis groups (15.0 vs. 15.1). Males showed a significantly higher prevalence of halitosis than females (24.5% vs. 22.7%, respectively, P < 0.001). Obesity, rural residence compared to city residence, poor health status, high stress, and low economic level demonstrated positive relations with halitosis (P < 0.001). Several dietary habits also related to halitosis. The frequent intake of alcohol, smoking, soda, fast food, instant noodles, and confections was associated with halitosis (P < 0.001). In contrast, the infrequent intake of fruit and vegetables was related to halitosis (P < 0.001) (Table 1).

**Table 1:** General Characteristics of Halitosis and Normal Participants.

	Normal Participants	Halitosis Participants	P-value
Number			
N	274,304	84,959	
%	76.4	23.6	
Mean Age (year)	15.0	15.1	<0.001*
Sex (%)			<0.001†
Male	75.5	24.5	
Female	77.3	22.7	
Obesity (%)			<0.001‡
Underweight	76.7	23.3	
Healthy	77.0	23.0	
Overweight	73.0	27.0	
Obese	69.6	30.4	
Region (%)			<0.001‡
Large City	76.6	23.4	
Small City	76.3	23.7	
Rural Area	75.4	24.6	
Subjective Health (%)			<0.001‡
Very good	83.6	16.4	
Good	78.1	21.9	
Normal	72.0	28.0	
Bad	62.2	37.8	
Very Bad	58.6	41.4	
Stress (%)			<0.001‡
Severe	68.6	31.4	
Moderate	72.4	27.6	
Mild	78.5	21.5	
A little	83.8	16.2	
No	86.6	13.4	
Economic Level (%)			<0.001‡
Highest	82.1	17.9	
Middle High	79.9	20.1	
Middle	77.9	22.1	
Middle Low	69.1	30.9	
Lowest	64.8	35.2	
Alcohol (%)			<0.001‡
0 day a month	76.9	23.1	
1–5 days a month	74.1	25.9	
6–19 days a month	74.4	25.6	
≥ 20 days a month	74.7	25.3	
Smoking (%)			<0.001‡
0 day a month	76.8	23.2	
1–5 days a month	73.4	26.6	
6–19 days a month	73.3	26.7	
≥ 20 days a month	72.4	27.6	
Fruit (%)			<0.001‡
≥7 times a week	79.4	20.6	
3–6 times a week	77.3	22.7	
1–2 times a week	74.6	25.4	
0 time a week	71.3	28.7	
Soda drink (%)			<0.001‡
≥7 times a week	75.0	25.0	
3–6 times a week	74.3	25.7	
1–2 times a week	76.8	23.2	
0 time a week	77.2	22.8	
Fast food (%)			<0.001‡
≥7 times a week	74.0	26.0	
3–6 times a week	72.8	27.2	
1–2 times a week	76.4	23.6	
0 time a week	77.4	22.6	
Instant noodle (%)			<0.001‡
≥7 times a week	72.7	27.3	
3–6 times a week	73.5	26.5	
1–2 times a week	76.9	23.1	
0 time a week	77.9	22.1	
Confectionary (%)			<0.001‡
≥7 times a week	74.2	25.8	
3–6 times a week	75.3	24.7	
1–2 times a week	77.2	22.8	
0 time a week	77.4	22.6	
Vegetable (%)			<0.001‡
≥7 times a week	77.5	22.5	
3–6 times a week	76.4	23.6	
1–2 times a week	74.0	26.0	
0 time a week	70.9	29.1	

* Paired T-test, Significance at P < 0.05.

^†^ Chi-square test, Significance at P < 0.05.

^‡^ Linear by linear association, Significance at P < 0.05

When estimated by simple logistic regression analysis with complex sampling, older age (OR = 1.05, 95% CI = 1.04–1.05, P < 0.001) and male gender were related to halitosis (OR = 1.09, 95% CI = 1.06–1.11, P < 0.001) (Table 2). Compared to healthy weight, overweight and obese subjects showed higher OR for halitosis (OR = 1.23, 95% CI = 1.19–1.26 and OR = 1.47, 95% CI = 1.41–1.55, respectively, P < 0.001), while being underweight was not associated with halitosis. Subjective health status was associated with halitosis, with OR ranges from 1.45 to 3.76 compared to ‘very good’ health status (95% CI = 1.42–1.49 and 3.31–4.28, respectively, P < 0.001). Compared with the no stress group, the severe stress group displayed an odds ratio of 2.94 (95% CI = 2.73–3.15, P < 0.001). Lower economic level was related to halitosis (OR = 2.47, 95% CI = 2.35–2.60, P < 0.001). Intake of alcohol, smoking, fast food or instant noodles more than 1 time per week and the intake of soda or confections more than 3 times per week were associated with a higher prevalence of halitosis (P < 0.001). Conversely, infrequent intake of fruits and vegetables was related to halitosis (P < 0.001).

**Table 2:** Odd ratio of possible risk factors for halitosis using simple and multiple logistic regression analysis with complex sampling.

Related Factors	Simple			Multiple		
	OR	95% CI	P-value	AOR	95% CI	P-value
**Personal Factors**						
Mean Age (year)	1.05	1.04–1.05	<0.001*	1.05	1.04–1.05	<0.001*
Sex			<0.001*			<0.001*
Male	1.09	1.06–1.11		1.21	1.19–1.24	
Female	1			1		
Obesity			<0.001*			<0.001*
Underweight	1.02	0.98–1.05		0.91	0.88–0.95	
Healthy	1			1		
Overweight	1.23	1.19–1.26		1.2	1.17–1.24	
Obese	1.47	1.41–1.55		1.37	1.31–1.44	
Subjective Health			<0.001*			<0.001*
Very good	1			1		
Good	1.45	1.42–1.49		1.37	1.34–1.41	
Normal	2	1.95–2.05		1.72	1.68–1.77	
Bad	3.15	3.04–3.27		2.44	2.35–2.53	
Very Bad	3.76	3.31–4.28		2.56	2.24–2.92	
Stress			<0.001*			<0.001*
Severe	2.94	2.73–3.15		2.05	1.91–2.20	
Moderate	2.46	2.29–2.63		1.9	1.77–2.04	
Mild	1.76	1.64–1.89		1.49	1.38–1.59	
A little	1.24	1.15–1.33		1.14	1.06–1.23	
No	1			1		
**Socioeconomic Factors**						
Region			<0.001*			0.05
Large City	1			1		
Small City	0.99	0.97–1.01		0.98	0.96–0.99	
Rural Area	1.07	1.03–1.11		1.01	0.98–1.04	
Economic Level			<0.001*			<0.001*
Highest	1			1		
Middle High	1.15	1.10–1.20		1.09	1.05–1.14	
Middle	1.29	1.24–1.34		1.15	1.11–1.20	
Middle Low	2.04	1.96–2.13		1.67	1.60–1.74	
Lowest	2.47	2.35–2.60		1.85	1.75–1.94	
**Dietary Habits**						
Alcohol			<0.001*			0.024*
0 day a month	1			1		
1–5 days a month	1.16	1.13–1.19		1.02	0.99–1.05	
6–19 days a month	1.16	1.11–1.21		0.96	0.91–1.00	
≥ 20 days a month	1.11	1.02–1.21		0.93	0.86–1.02	
Smoking (%)			<0.001*			0.501
0 day a month	1			1		
1–5 days a month	1.17	1.12–1.23		1.02	0.97–1.07	
6–19 days a month	1.22	1.15–1.30		1.05	0.98–1.12	
≥ 20 days a month	1.24	1.20–1.29		1	0.97–1.04	
Fruit			<0.001*			<0.001*
≥7 times a week	1			1		
3–6 times a week	1.15	1.13–1.18		1.07	1.04–1.10	
1–2 times a week	1.32	1.29–1.36		1.12	1.09–1.15	
0 time a week	1.58	1.53–1.63		1.22	1.18–1.26	
Soda drink			<0.001*			<0.001*
≥7 times a week	1.1	1.05–1.15		0.91	0.87–0.96	
3–6 times a week	1.17	1.14–1.20		1.01	0.98–1.03	
1–2 times a week	1.01	0.99–1.03		0.96	0.94–0.98	
0 time a week	1			1		
Fast food			<0.001*			<0.001*
≥7 times a week	1.18	1.20–1.26		1.01	0.94–1.09	
3–6 times a week	1.3	1.26–1.33		1.15	1.12–1.19	
1–2 times a week	1.07	1.05–1.09		1.05	1.03–1.07	
0 time a week	1			1		
Instant noodle			<0.001*			<0.001*
≥7 times a week	1.34	1.26–1.42		1.17	1.10–1.25	
3–6 times a week	1.26	1.23–1.29		1.12	1.09–1.15	
1–2 times a week	1.06	1.04–1.08		1.04	1.02–1.06	
0 time a week	1			1		
Confectionary			<0.001*			<0.001*
≥7 times a week	1.19	1.14–1.24		1.17	1.10–1.19	
3–6 times a week	1.12	1.09–1.15		1.1	1.08–1.13	
1–2 times a week	1.02	0.99–1.04		1.03	1.00–1.06	
0 time a week	1			1		
Vegetable			<0.001*			<0.001*
≥7 times a week	1			1		
3–6 times a week	1.07	1.05–1.09		1	0.98–1.02	
1–2 times a week	1.22	1.19–1.25		1.08	1.05–1.11	
0 time a week	1.43	1.37–1.49		1.19	1.14–1.25	

* Significance at P < 0.05

All of the analyzed variables showed significant associations with halitosis. Therefore, reciprocally considering these covariates was prerequisite. Therefore, we performed a multiple logistic regression analysis with complex sampling (Table 2).

The results showed that old age, male gender, overweight or obese, poor health status, stress, and lower economic status were related to halitosis (P < 0.001). Compared to living in a large city, living in a small city was related to less halitosis (adjusted OR [AOR] = 0.98, 95% CI = 0.96–0.99, P = 0.050); however, no significant relation with living in a rural area was found.

Alcohol and smoking did not show significant associations with halitosis. The relation between soda consumption and halitosis was inconsistent. Compared to the no soda group, the OR of soda consumption ≥ 7 times per week was 0.91 (95% CI = 0.87–0.96, P < 0.001); however, the OR of soda intake 3–6 times per week was 1.01 (95% CI = 0.94–0.98, P < 0.001). Frequent intake of fast food was associated with halitosis (AOR = 1.15, 95% CI = 1.12–1.19, P < 0.001); however, fast food intake more than 7 times per week did not a show significant correlation with halitosis. Instant noodle and confection consumption showed a positive correlation with halitosis (AOR = 1.17, 95% CI = 1.10–1.25, P < 0.001). In contrast, fruit intake showed a negative correlation with halitosis (AOR = 1.22, 95% CI = 1.18–1.26, P < 0.001 for 0 times per week compared to ≥ 7 times per week). Vegetable intake also showed a negative correlation with halitosis (AOR = 1.19, 95% CI = 1.14–1.25, P < 0.001 for 0 times per week compared to ≥ 7 times per week) but lost significant correlation when eaten more than 3 times per week.

## Discussion

The prevalence of halitosis in Korean adolescents was 23.6% in the current study. We also identified various factors associated with halitosis. These associated factors included not only dietary factors but also demographic factors such as old age, sex, overweight or obese and psychosocial factors including subjective health status, stress and economic levels. In particular, we investigated whether the intake of various types of food affected halitosis. The data showed that fast food, instant noodle and confection intake was positively related to halitosis, while fruit and vegetable intake showed negative correlations with halitosis. Thus far, to our knowledge, no other study has examined the effects of each type of food on halitosis.

Halitosis originates from oral sources in most cases [1]. The most common intraoral causes of halitosis are chronic bacterial infection coating the tongue or associated periodontal diseases, including gingivitis, periodontitis, stomatitis and xerostomia [5]. The wide range of oral anaerobic bacteria may produce odorous materials, VSCs, diamines, and phenyl compounds [14]. In addition to infectious causes, mucosal ulceration, impacted food or debris and tongue coating are related to halitosis [14]. Dietary habits can influence halitosis by both infectious and non-infectious mechanisms.

Several studies have reported that diet, smoking and drinking are associated with halitosis [8, 14, 15]. Frequent ingestion of sugar snacks may result in dental caries, a well-known cause of halitosis [15, 16]. Fast foods, instant noodles, and confections are associated with obesity, and gastrointestinal diseases including gastric reflux raise the gastric contents of gastric acid and digestive enzymes to the upper digestive tract and oral cavity [17]. Therefore, dental caries and gastric reflux related to fast foods, instant noodles and snacks might provoke halitosis. Although fast food intake more than 7 times a week did not show a significant correlation with halitosis, this result may be because fast food intake was overwhelmed by other covariates that plague this group. In contrast, vegetable and fruits with high fiber contents promote gastric emptying. Moreover, certain vegetable extracts such as green tea extract and vegetable acetone powder have been reported to effectively remove VSCs and to reduce gingivitis, subsequently reducing halitosis [18, 19]. Smoking did not show a significant correlation with halitosis in the present study by multiple regression analysis. Statistical significance was most likely difficult to achieve due to the relatively small portion of smokers in our study population.

We identified several psychosocial factors including subjective health status, stress, and economic level that were associated with halitosis. Previous studies have reported cultural or ethnic differences in the perception and incidence of halitosis [20]. Moreover, several studies have suggested that socio-economic inequalities in oral health exist among adolescents [21–23]. Self-reported oral symptoms may reflect social gradients, and these gradients can be partially explained by psychosocial factors [24]. In particular, halitosis could be more easily perceived by stressed or nervous subjects than by stress-free subjects, even if halitosis has not reached a threshold level to be objectively detected. When halitosis is objectively measured, this halitosis with a low threshold value can be obscured by pseudo-halitosis.

Alternatively, stress may have implications for halitosis through increasing the VSCs via mouth breathing [25]. This stressful situation diminishes salivary flow, which leads to the degradation of retained proteins in the mouth, and consequently VSCs could increase. However, during stressful situations such as premenstrual syndrome, VSCs increase without a decrease in salivary flow [25]. Hormonal changes that affect oral bacterial number and mucous desquamation and weaken the host immune system through salivary IgA concentration may contribute to the increase the oral VSCs, even in the presence of normal salivary flow [26, 27].

This study had specific limitations. Few verified studies on halitosis exist due to the subjective characteristics of halitosis. First, halitosis is a subjective symptom and is therefore hard to objectively measure [5]. We used self-reported halitosis, which is often unreliable. Discriminating halitosis from halitophobia, which is a delusional disorder that is considered a type of olfactory reference syndrome (ORS), and from pseudo-halitosis, which refers conditions that are characterized with a subjective sensation of malodor with an inability to provide objective evidence, was difficult [28, 29]. However, objective measurements using a portable VSC detector, halimeter, or gas chromatography to assess halitosis quantitatively are generally insensitive and often unavailable under clinical circumstances [30–32]. Moreover, several previous studies have suggested that a strong correlation exists between self-assessed halitosis and objectively measured halitosis [33–35]. Although some conflicts in the diagnostic values exist, approximately 72.1 to 93.9% of patients with subjective halitosis were able to demonstrate their halitosis by objective measures [33, 34]. The converse is also true; the majority of subjects with detectable halitosis by halimeter or organoleptic assessments also subjectively recognized their halitosis [35]. Notably, our study population was conducted on middle and high school students, who generally have good perception abilities. Therefore, the diagnostic accuracy of subjective halitosis was predicted to be higher than for the general population.

Second, selection bias often occurs because affected subjects are often reluctant to participate in a survey because they feel shame for their breath odor. This problem is significant because it make it hard to survey for halitosis. We minimized this selection bias using a representative population-based study group. Finally, several types of confounding factors exist that we did not consider, such as dental caries, dental treatment history, and other oral conditions. To minimize confounding effects, these oral factors were indirectly considered by the survey as part of the subjective health status.

## Conclusion

In the current study, we identified a considerable number of adolescents with halitosis. Various demographic, psychosocial and dietary factors contribute to halitosis. Based on these results, when a clinical practitioner evaluates patients with halitosis, they should consider the demographic and psychosocial status of the patients, as well as the comprehensive physical examination and history, focusing on intra-oral, extra-oral, and systemic diseases that are associated with halitosis. Interviewing a patient regarding lifestyle factors such as diet, smoking, stress and social economic status (SES) will be helpful for elucidating a patient’s hidden causes of halitosis.
